# Crystallisation Route of Tin-Based Bearing Alloys in Static and Centrifugal Casting

**DOI:** 10.3390/ma19204408

**Published:** 2026-10-20

**Authors:** Marcin Kondracki, Andrzej Studnicki, Natalia Przyszlak, Jacek Suchoń

**Affiliations:** Department of Foundry Engineering, Faculty of Mechanical Engineering, Silesian University of Technology, Towarowa 7, 44-100 Gliwice, Poland; andrzej.studnicki@polsl.pl (A.S.); natalia.przyszlak@polsl.pl (N.P.); jacek.suchon@polsl.pl (J.S.)

**Keywords:** centrifugal casting, gravity die casting, crystallisation, structure, tin-based alloys, plain bearings

## Abstract

In this paper, the authors analyse the crystallisation of bearing alloys used for heavy-duty plain bearings. The composition consists of tin, antimony, copper, and small additions of modifying elements. All the constituents are present in various amounts in the matrix and primary intermetallic phases. Due to a wide range of solidification factors, like cooling rate and pressure, these can change the sequence of crystallisation or lead to segregation of structural components. The present study compares the equilibrium crystallisation of the studied alloys, calculated with Thermo-Calc software, with the experimental results. Data was acquired using the TDA method for alloys poured into standard sand cups as well as into metallic probes of the same geometry, in cold and heated states. The final experiment was conducted with crystallisation analysis during centrifugal casting of alloys poured into steel and cast-iron model bearing housings with different initial temperatures. The authors made an effort to compare and describe the route of crystallisation of the alloys and the influence of the technological process on their structures. The studies also included an extended examination of the structure of the alloys and phenomena connected with the possible segregation of structural components using SEM and optical microscopy.

## 1. Introduction

Tin-based plain bearing alloys, commonly referred to as tin babbitts, represent one of the most traditional and still widely used groups of bearing materials. Their unique combination of a relatively soft matrix with evenly distributed hard phases provides excellent tribological properties, including a low friction coefficient, outstanding compatibility with steel journals, good embeddability of foreign particles, and conformability. These characteristics make them particularly suitable for medium- and high-speed hydrodynamic operation under varying loads. They are especially popular in turbines for the power engineering industry [1,2,3].

The microstructure of tin-based bearing alloys is the result of a complex crystallisation process. In typical Sn-Sb-Cu alloys, solidification begins with the formation of primary intermetallic compounds—needle-like or rosette Cu_6_Sn_5_ (η phase) and cubic SnSb large crystals (β phase)—followed by the crystallisation of a soft, tin-rich solid solution mixed with fine β crystals, similar to eutectic structures. The size, morphology, and distribution of these hard phases decisively determine the mechanical and tribological properties of the finished bearing. Too-coarse crystals reduce the fatigue strength and may cause scoring, while a fine and uniform distribution significantly improves load-carrying capacity and wear resistance [3,4]. Although the alloys react to increased cooling rates in this range, too-high values of this factor can lead to segregation due to the wide temperature range of solidification. Additionally, a poorly performed metallurgical process can lead to the inheritance of an improper structure, as the intermetallic crystals differ significantly in size and thermophysical properties [5,6,7].

Despite their excellent bearing properties, tin-based Babbitt alloys have several important limitations. One of the main limitations is their relatively low resistance to softening at elevated temperatures. Typically, above approximately 130–150 °C, these alloys begin to lose mechanical strength, which limits their use in high-performance engines and machinery. To improve their high-temperature performance, additional alloying elements, such as cadmium, arsenic, or nickel, may be introduced. These elements can modify the solidification sequence, affect the volume fraction and stability of hard phases, and improve the retention of mechanical strength at elevated temperatures [8,9,10,11,12].

An important aspect that remains insufficiently understood is the influence of rapid cooling, forced liquid metal flow, and dynamic conditions during solidification on the crystallisation process. Centrifugal forces, vibrations, and increased heat-extraction rates can substantially alter nucleation rates, crystal growth morphology, and the distribution of intermetallic phases compared with those observed under equilibrium conditions. Understanding these relationships during the centrifugal casting of tin-based bearing alloys was the primary motivation for the present study. Bearings for power plant turbines are typically cast into one of two types of housings: lightweight steel inserts or massive cast-iron housings. In both cases, the housings are preheated, and their contact surfaces are prepared using tin and various types of flux to ensure proper bonding between the housing and the cast bearing-alloy layer. Because the two types of housings provide different thermal conditions, a comprehensive analysis must account for the most important influencing factors, particularly since each type is also associated with different material quality issues. In the present study, simplified steel and cast-iron moulds were used to represent these two cases. Based on dimensional analysis, moulds with geometric and thermal similarity to the industrial components were designed for the experiments. This approach represents one of the first attempts to monitor the solidification process of bearing alloys under conditions representative of centrifugal casting.

## 2. Materials and Methods

The present study investigated two alloys used for plain bearings. The standard SnSb11Cu6 alloy, specified in ISO 4381:2011 [13], was used as a reference material in the static part of the experiment. The main focus was on the alloy commercially designated V738, whose chemical composition differs primarily in the content of alloying elements that extend its operating temperature range, mainly by strengthening the matrix at elevated temperatures. This alloy is commonly referred to as nickel-enhanced Babbitt and also contains cadmium and arsenic. The chemical compositions of both alloys are presented in Table 1. Chemical analysis was performed using a SpectroMaxx 6Gen spectrometer manufactured by SPECTRO Analytical Instruments GmbH (Kleve, Germany). Fresh material was used for each melt investigated in this study, unless otherwise stated.

The alloys were melted in a laboratory-scale, medium-frequency coreless induction furnace using a cast-iron crucible with a capacity of 4 kg. The pouring temperature was set to 510 °C for the SnSb11Cu6 alloy and 530 °C for the V738 alloy. After melting, ammonium chloride was used as a refining agent at a concentration of 1 wt.%. The crystallisation behaviour of the alloys was investigated using thermal and derivative analysis (TDA). Samples were poured into various cups and moulds before and after the refinement treatment. Commercial Quik-Cup probes (Heraeus Electro-Nite, Sosnowiec, Poland) were used. A second specimen from each melt was poured into a cast-iron cup of the same geometry, preheated to an initial temperature of 200 °C. Temperature was recorded using K-type thermocouples connected to an industrial multichannel data acquisition unit.

**Figure 1:**
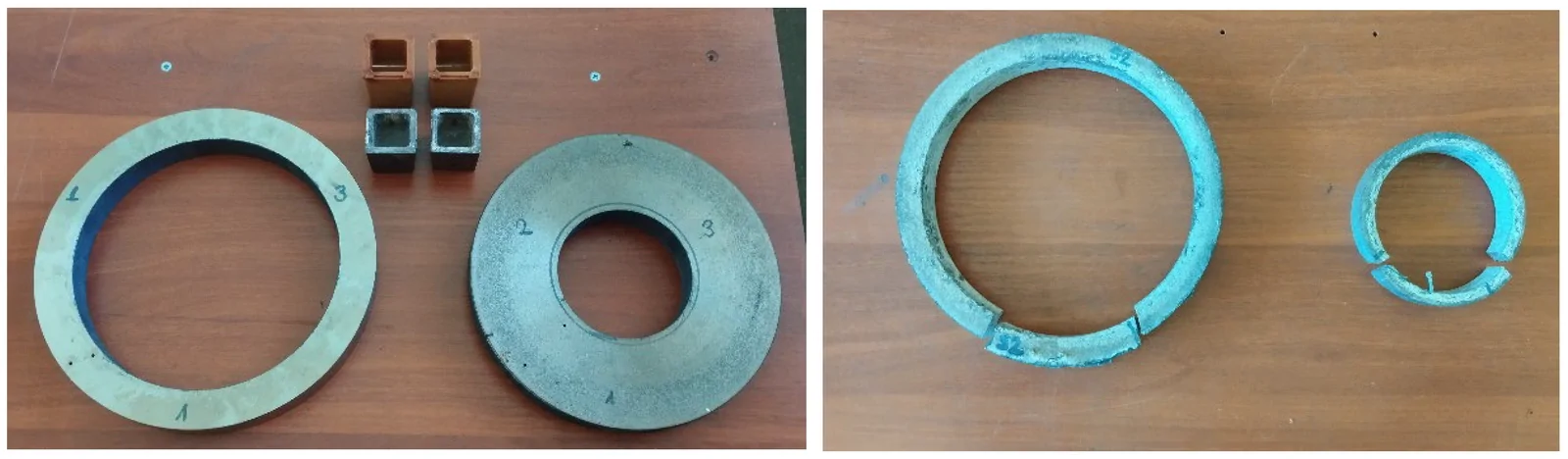
Moulds and castings used in the experiment: moulds for static and centrifugal casting (**left**); castings with sections cut for microstructural examination (**right**). A detailed description is provided in the text, and the dimensions are listed in Table 2.

**Figure 2:**
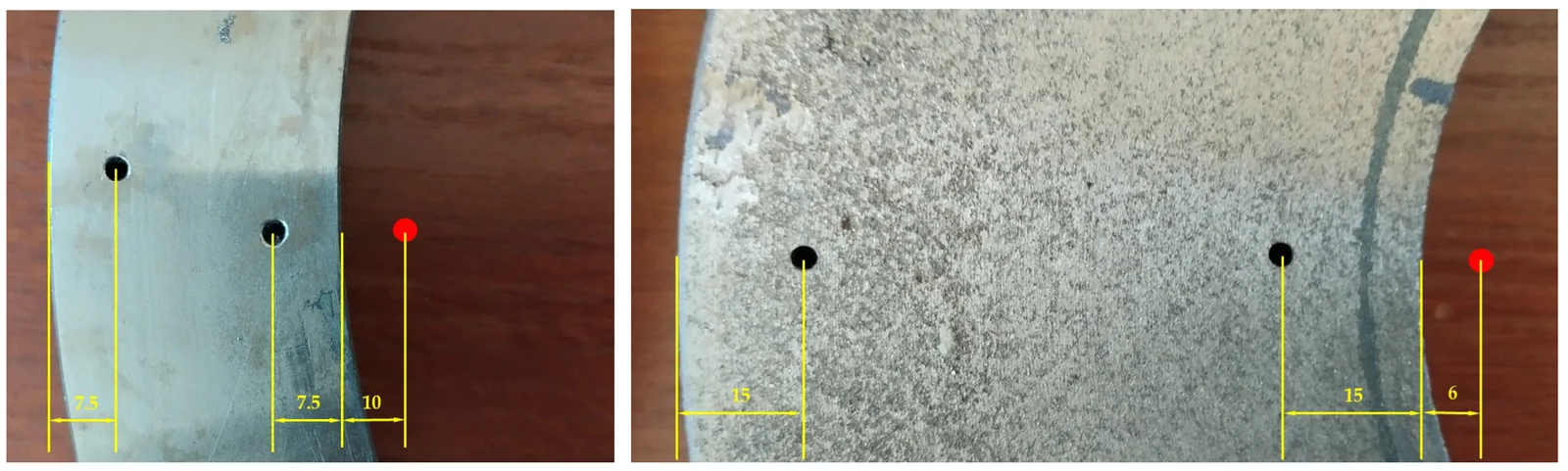
Placement of thermocouples in the steel mould (**left**) and cast-iron mould (**right**). The dimensions indicate the positions of the 2.5 mm-diameter holes for the thermocouples relative to the inner and outer mould surfaces. The red dot indicates the nominal position of the thermocouples inside the castings. All thermocouples were positioned at the mid-thickness of the casting/mould section (17.5 mm deep).

The second part of the experiment investigated the solidification of the V738 alloy during centrifugal casting, together with the heat balance in the casting–mould system. All the equipment used in this part of the study was designed and prepared in the Department of Foundry Engineering. Two types of mould were used. The first consisted of a relatively thin-walled ring made of plain low-carbon steel with a ferritic microstructure, while the second was made of cast iron and had thicker walls. The geometric characteristics of the moulds were selected to reflect the features of plain bearing housings used in industrial applications. The shapes and geometric features of both systems are shown in Figure 1 and summarised in Table 2. The moulds were closed with covers made of fibre-based plates and insulated with a 5 mm-thick refractory sheet (Sibral Standard, quartz–alumina fibre material). Four thermocouples were installed in each mould. Two were positioned inside the mould cavity, 10 mm from the inner mould wall, to record the temperature of the solidifying casting. The other two were placed in the mould ring, near its inner and outer surfaces. The specific measurement arrangements differed between the two moulds and are shown in Figure 2.

The measurement system consisted of a transmitter mounted on the bottom plate of the centrifugal casting machine and a receiver connected to a PC. Both units were based on Arduino-compatible microcontroller boards incorporating a 2.4 GHz wireless communication module and MAX31855 thermocouple-to-digital converters (Maxim Integrated Products, San Jose, CA, USA), one for each measurement channel. This architecture, together with dedicated software, enabled temperature recording at sampling intervals of 42–43 ms, corresponding to a sampling frequency of approximately 23 Hz. The centrifugal casting machine, located in the Department of Foundry Engineering, was driven by a 1.1 kW asynchronous electric motor powered by a high-frequency inverter (three-phase, 4 kW; Hufeng Electronics, Suzhou, China), which was used to regulate the rotational speed. The motor and spindle were connected by a single-belt transmission.

Each type of mould was used in two casting trials. In the first trial, the mould rings were preheated to an initial temperature in the range of 250–300 °C, with each mould tested in a separate experiment. In the second trial, the moulds were used at room temperature. Preheating was carried out in an electrically heated resistance chamber furnace MDM F400 (Mario Di Maio, Gerenzano, Italy). Once the temperature reached 300 °C, the rings were held in the furnace for one hour. The temperature of each ring was checked using a thermal imaging camera. However, owing to the mounting procedure and differences in the geometric and physical properties of the rings, the actual initial temperatures recorded by the thermocouples were lower than the furnace temperature. The actual values can be determined from the TDA diagrams.

The metallurgical procedure was the same as that used in the previous part of the experiment. The rotational speed for each mould was selected according to industrial guidelines, considering the diameter of the casting free surface. The rotational speed was set to 450 rpm for the steel mould and 600 rpm for the cast-iron mould to account for differences in mould geometry and maintain comparable casting conditions. After each experiment, the castings were removed from the moulds and sectioned for metallographic examination. Samples were prepared for optical microscopy and scanning electron microscopy (SEM) with energy-dispersive X-ray spectroscopy (EDS). Micrographs were acquired along the casting cross-sections to compare the microstructures in different regions and under different casting conditions. Microstructural examination was performed using Nikon Eclipse LV150N (Nikon, Tokyo, Japan) and Keyence VHX-S650E (Keyence Corporation, Osaka, Japan) optical microscopes. SEM and EDS analyses were carried out using a Phenom ProX microscope (Thermo Fisher Scientific, Waltham, MA, USA).

## 3. Results

**Figure 3:**
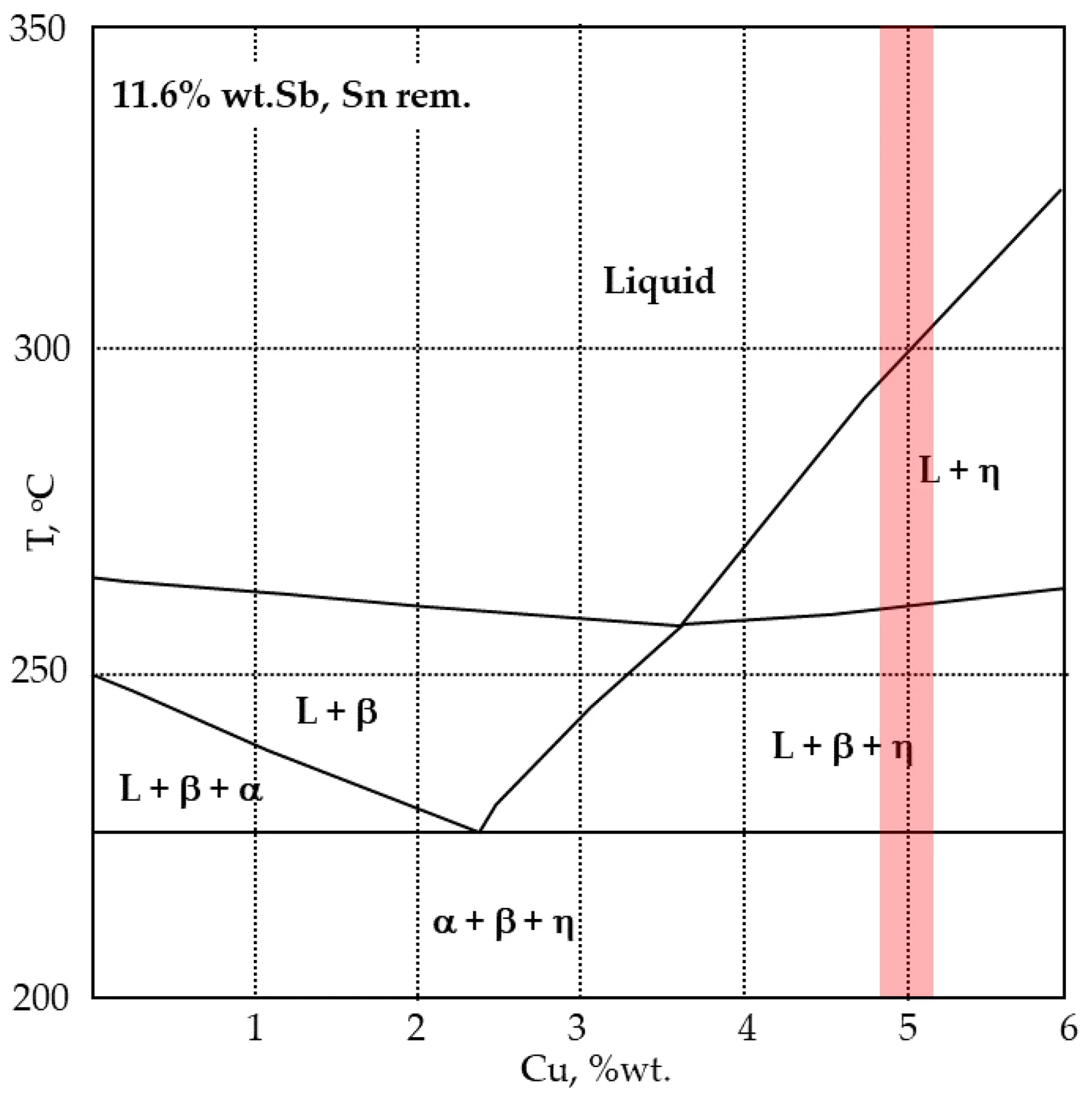
Section of the Sn–Sb–Cu ternary phase diagram at a constant Sb content of 11.6 wt.%, showing the effect of Cu content on phase transformations, with Sn as the balance element. The diagram was calculated using Thermo-Calc. The shaded region indicates the Cu content range of the alloys investigated in this study.

**Figure 4:**
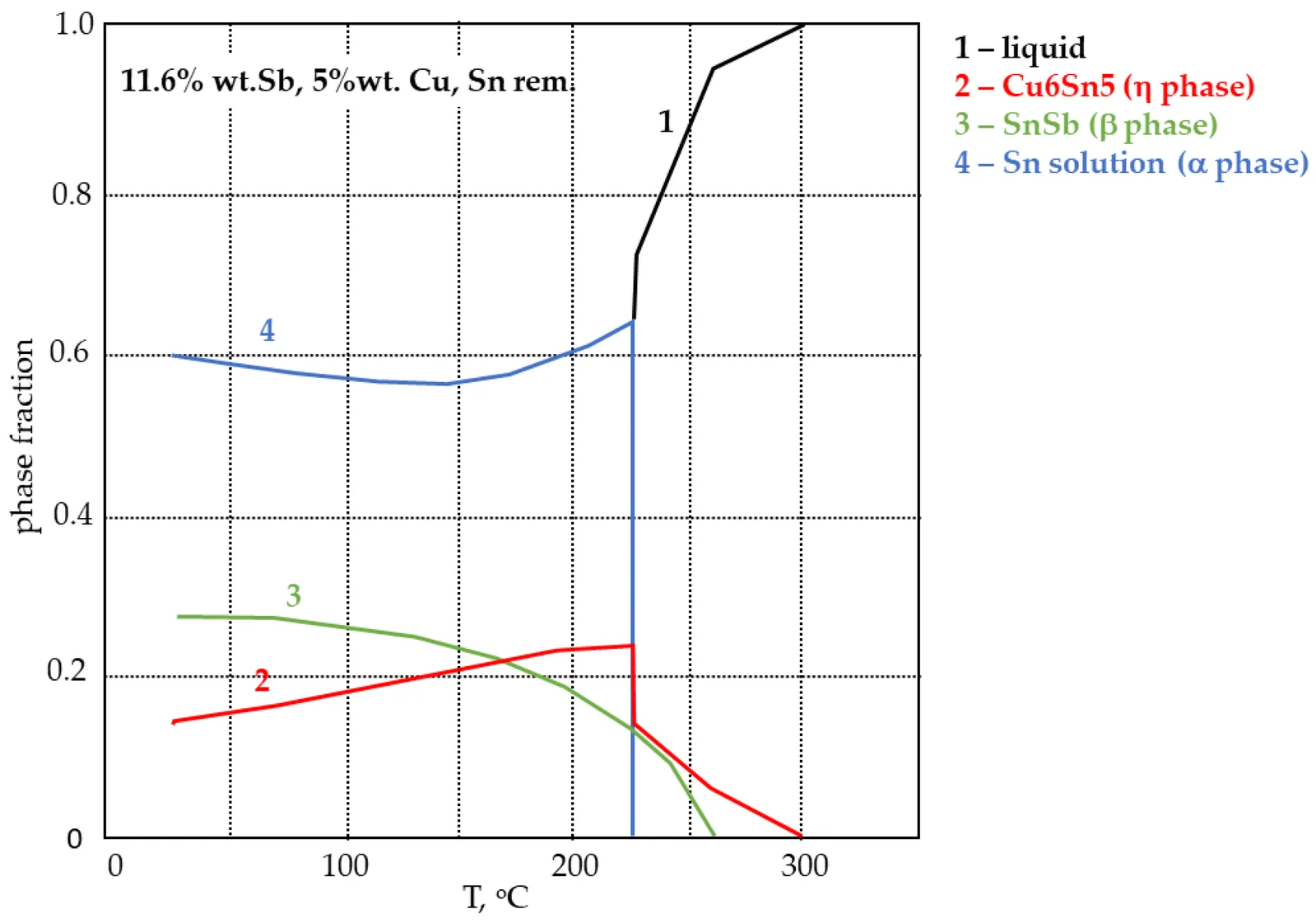
Phase fractions as a function of temperature for the SnSb11.6Cu5 alloy, calculated using Thermo-Calc software. The crystallisation sequence is described in detail in Table 3.

The experimental data consisted of TDA curves, micrographs, and phase identification results. These data provided complementary, indirect insights into the solidification process, including thermal effects during cooling and the resulting alloy microstructure. Their interpretation can be supported by established knowledge of the alloys and thermodynamic calculations. As the two alloys have similar chemical compositions, a simplified system containing only the three principal elements (Sn, Sb, and Cu) was modelled using Thermo-Calc software (version R) under equilibrium conditions. The most relevant results are presented in Figure 3 and Figure 4 and Table 3.

According to the thermodynamic analysis (Figure 3) and established knowledge of the alloy system, the primary phase to crystallise is Cu_6_Sn_5_ (the η phase), followed by the formation of primary SnSb crystals (the β phase). Solidification concludes with the formation of the matrix, which consists of a tin-rich solid solution and fine intermetallic crystals arranged in a eutectic-like structure. The evolution of the alloy chemical composition during solidification is presented in greater detail in Figure 4 and Table 3. According to the equilibrium calculations, solidification begins at 300 °C with the formation of the η phase. As the temperature decreases to 260 °C, the η-phase fraction increases to approximately 6 vol.%, while the liquid composition approaches the conditions required for the β phase to form. Both phases continue to crystallise, progressively changing the composition of the remaining liquid. At 230 °C, the system reaches another characteristic point at which the η-phase fraction increases more rapidly and crystallisation of the α phase begins. The near-constant temperature during further solidification down to the solidus indicates substantial latent heat release associated with the final transformation. The equilibrium calculations also predict changes in the relative phase fractions during secondary crystallisation.

Although the conditions in an actual casting differ significantly from those assumed under equilibrium, the calculations provide an indication of the likely primary phase to form, the relevant temperature ranges, and the types of thermal effects to identify in the TDA curves. The formation of both intermetallic phases is expected to produce sharp, triangular peaks, whereas the final solidification of the matrix is expected to appear as a plateau, consistent with the transformation mechanisms described above. It is also important to note that the thermal stability temperatures of both intermetallic phases, as indicated by the corresponding binary phase diagrams (the Sn–Cu diagram for the η phase and the Sn–Sb diagram for the β phase), are higher than the temperatures predicted by the calculations for the ternary system, particularly during the remelting of a previously prepared alloy.

**Figure 5:**
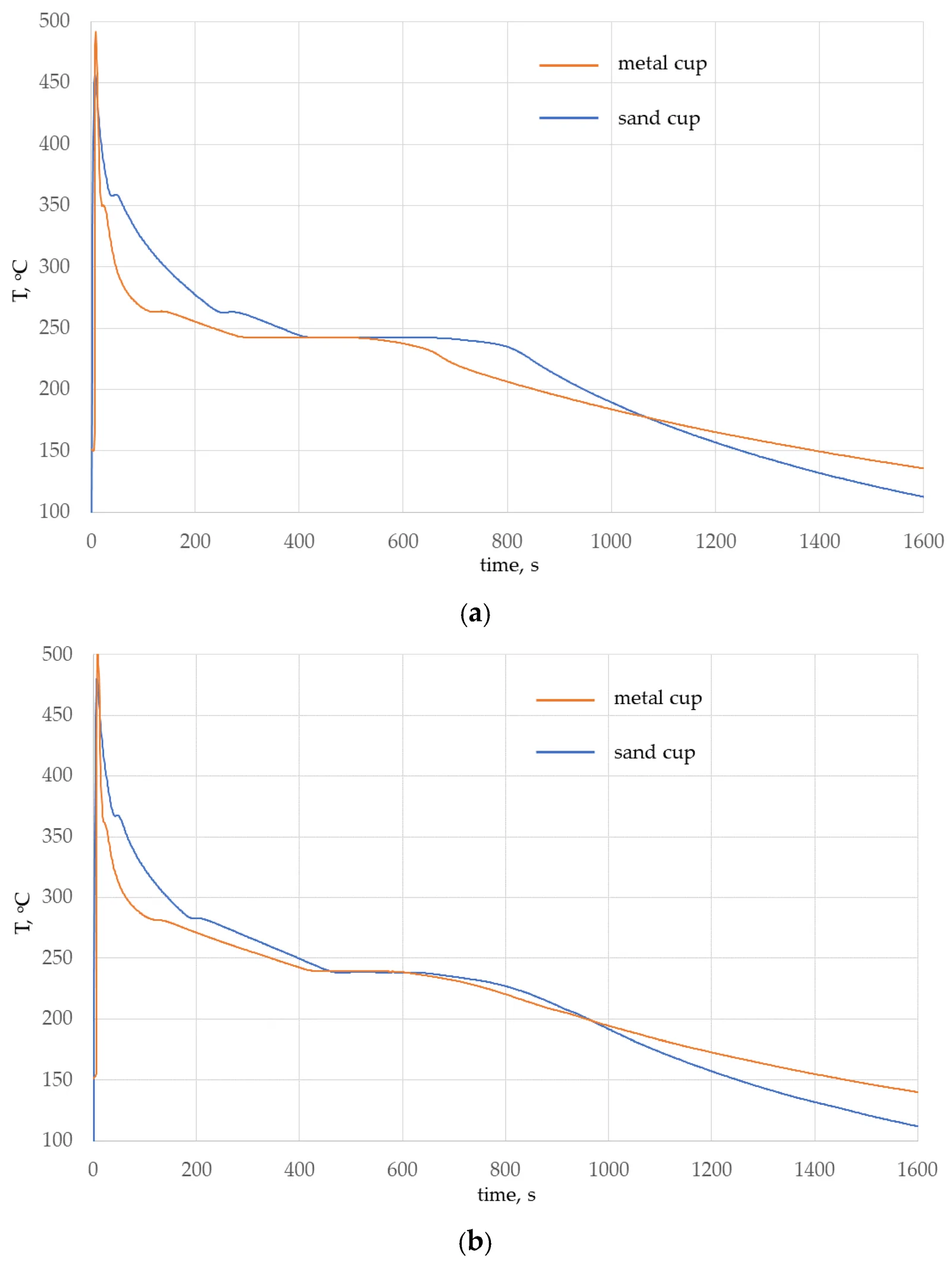
Thermal curves for the SnSb11Cu6 (**a**) and the V738 (**b**) alloys obtained using two types of TDA cups: a sand cup (Quik-Cup) and a cast-iron cup of the same geometry.

Figure 5 shows the temperature changes during the solidification of the studied alloys. The curves represent the refined alloys measured using different types of cups. As can be seen, the overall characteristics of the curves are similar. The main difference for both alloys lies in the initial temperature of the primary η-phase formation and the possible occurrence of undercooling. This effect is particularly evident for the SnSb11Cu6 alloy, for which nucleation is promoted by the higher cooling rate associated with the metal cup, resulting in the absence of undercooling. Another notable difference between the two alloys is the higher onset temperatures of both intermetallic phases in the V738 alloy. This may be attributed to differences in the Sn/Cu and Sn/Sb ratios, as well as to the inoculating effect of additional alloying elements. Furthermore, for a given alloy, the characteristic temperatures associated with β-phase formation and matrix solidification remain almost unchanged at different cooling rates, as represented by the different cups. These observations suggest that, among the three main solidification transitions, the formation of the primary η phase is the most sensitive to the effects of inoculation and cooling rate.

**Figure 6:**
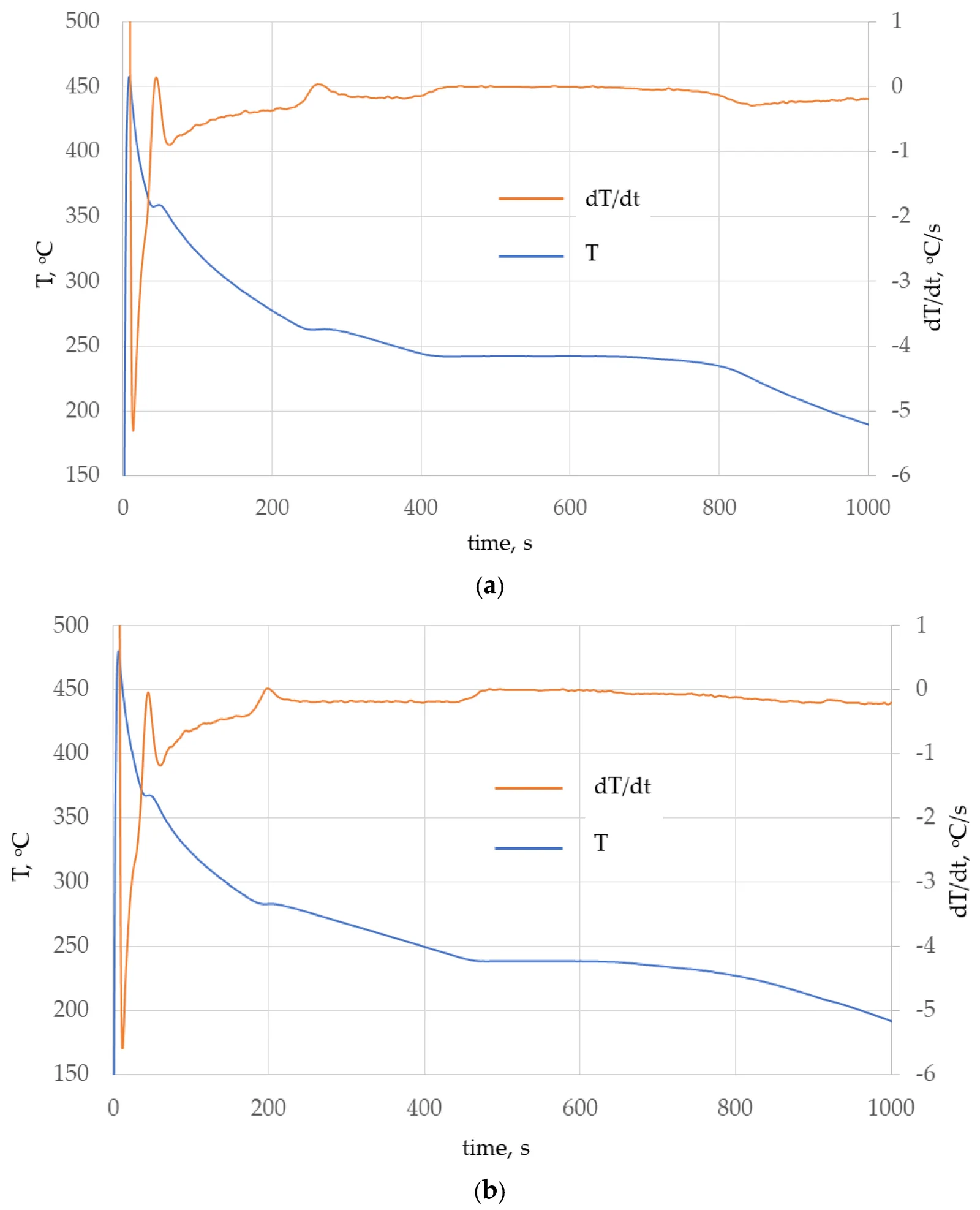
TDA curves for the SnSb11Cu6 (**a**) and V738 (**b**) alloys: pure alloy after refinement in a sand cup.

By adding the first derivative to the temperature–time curve (Figure 6), the complete thermal and derivative analysis (TDA) can be obtained. The resulting curves exhibit the shapes predicted for the consecutive solidification transitions. The first two thermal effects, visible in the first derivative of temperature with respect to time ((dT/dt)), have a triangular shape, whereas the third exhibits a characteristic plateau with a trapezoidal profile. The first derivative remains positive throughout all three transitions, suggesting that no significant undercooling associated with nucleation delay is observed.

**Figure 7:**
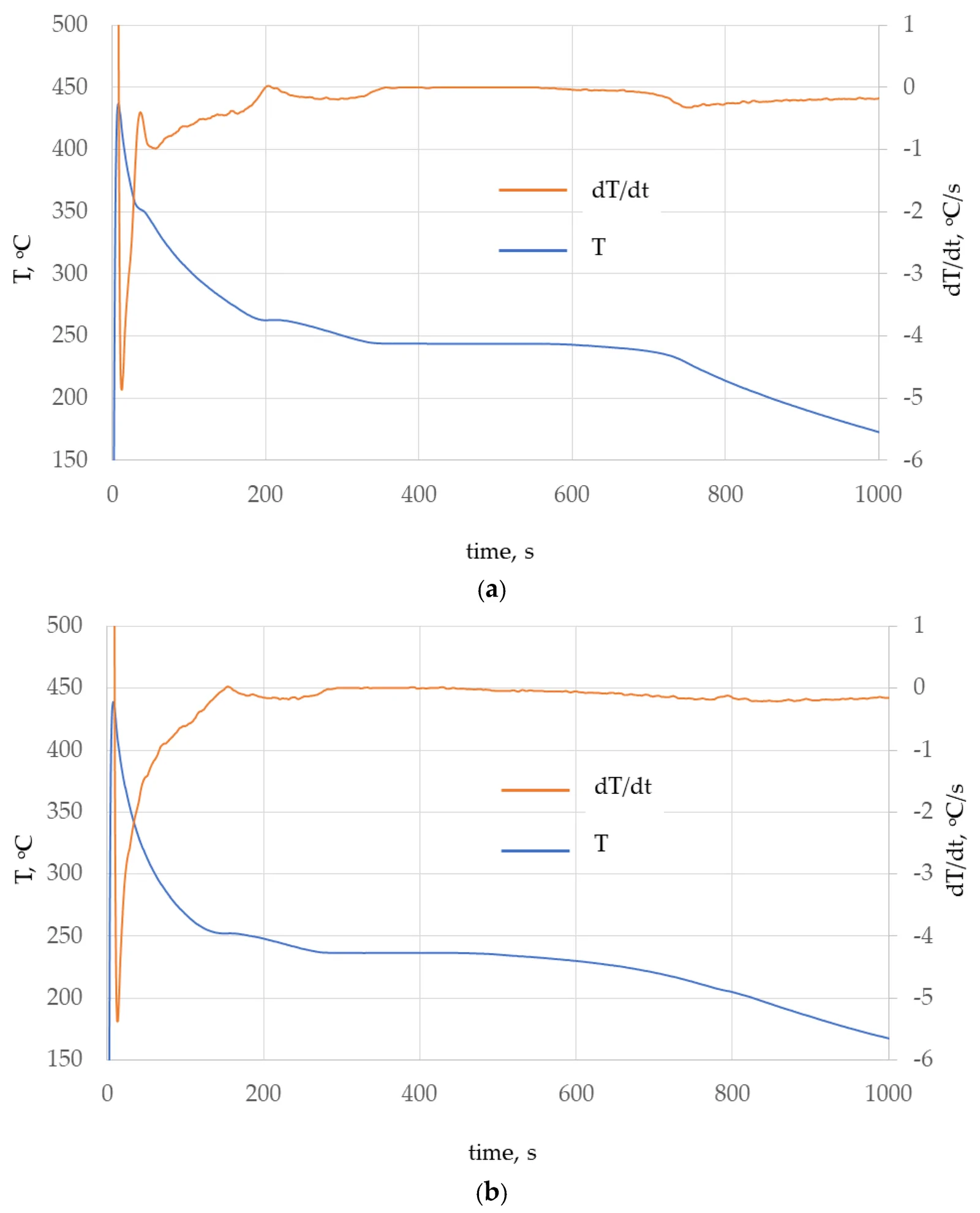
TDA curves for the SnSb11Cu6 alloy: alloy after several cycles before refinement (**a**) and reclaimed alloy before refinement (**b**), both in sand cups.

For the V738 alloy, the primary phase begins to crystallise without undercooling. For the other two transitions, the derivative reaches positive values, indicating different transformation kinetics compared with those of the primary phase. A further interesting comparison can be made between the TDA curves of the as-received alloy and those of material subjected to several remelting cycles or obtained through reclamation. The main qualitative differences are discussed below. Figure 7 presents the TDA curves for the SnSb11Cu6 alloy. Figure 7a shows the alloy after several remelting cycles, with refinement performed during each cycle. However, the final TDA measurement was carried out without refinement. Figure 7b shows the reclaimed alloy before the refinement treatment.

As shown in the figure, the thermal effect associated with the η phase is less pronounced and occurs at a lower temperature, without undercooling. The second thermal effect, associated with β-phase crystallisation, exhibits a positive derivative value, although its shape differs slightly from that observed in the reference material. In the material containing a high level of impurities, the thermal effect associated with the primary η phase is not clearly distinguishable. Instead, irregularities in the crystallisation curve indicate unstable η-phase formation within the 51–120 s interval. The second transition occurs at a lower temperature, which may be associated with changes in the alloy composition resulting from antimony oxidation. Changes are also observed during matrix solidification. An additional thermal effect appears at the end of the plateau, within the 770–800 s interval. Such effects are commonly attributed to impurities that promote the formation of low-melting-point eutectics during the final stages of solidification.

**Figure 8:**
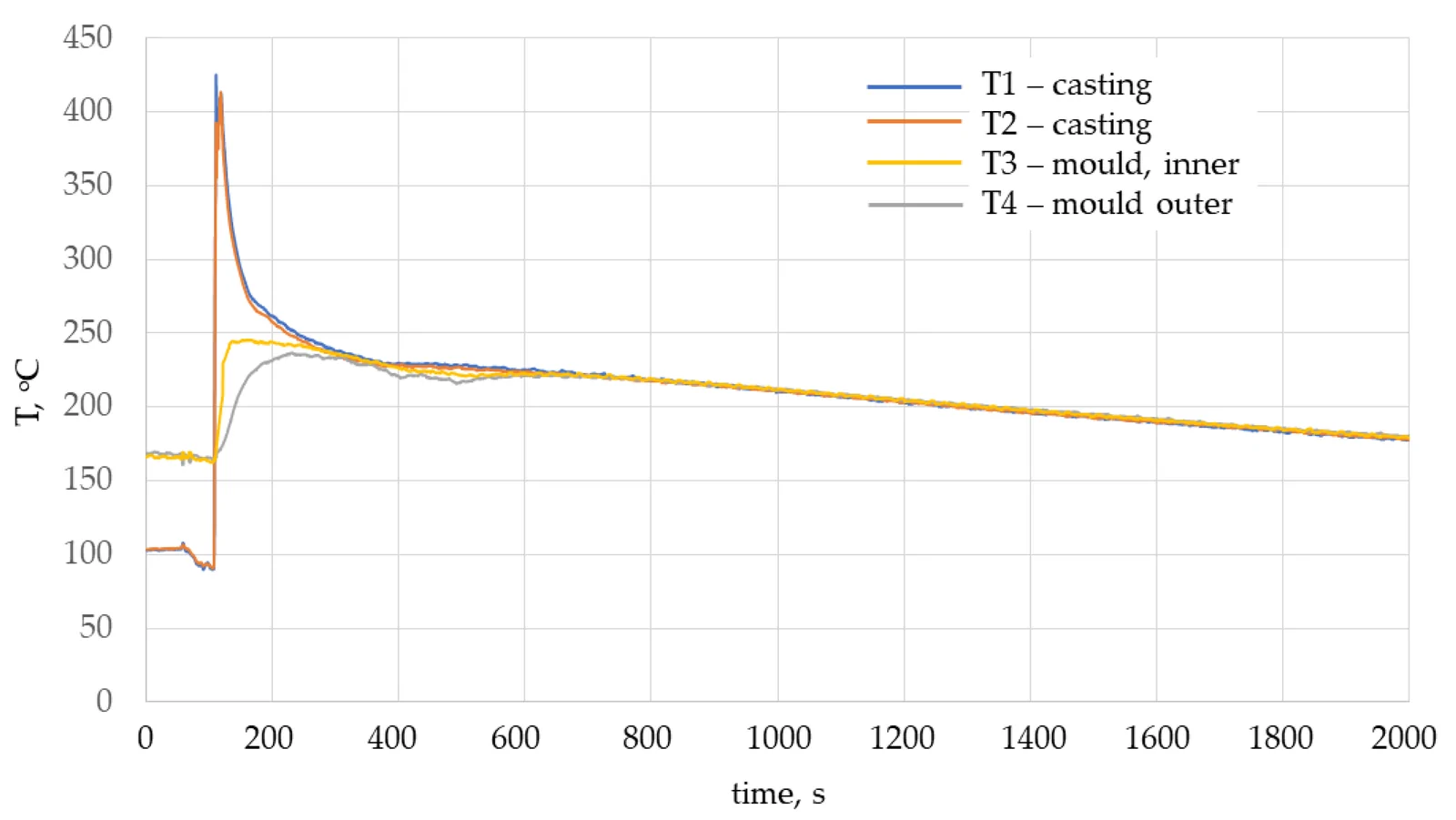
Thermal curves for the V738 alloy during centrifugal casting in a preheated steel mould.

The second part of the TDA experiment was conducted during centrifugal casting, with temperature recorded wirelessly throughout the process. The V738 alloy used in this part of the study was refined during each melting cycle. The molten alloy was poured into steel and cast-iron moulds under both preheated and unheated (room-temperature) conditions, resulting in four experimental configurations. Figure 8 presents the results obtained using the preheated steel mould. The first diagram contains four temperature curves: T1 and T2 represent measurements taken inside the casting, while T3 and T4 correspond to measurements within the mould ring. T3 represents the region closer to the mould cavity (inner surface), whereas T4 represents the region closer to the outer surface. After pouring, the outer surface of the mould was cooled using a water mist. This cooling was applied only during the primary crystallisation stage. The procedure was intended to reproduce cooling conditions representative of industrial practice.

**Figure 9:**
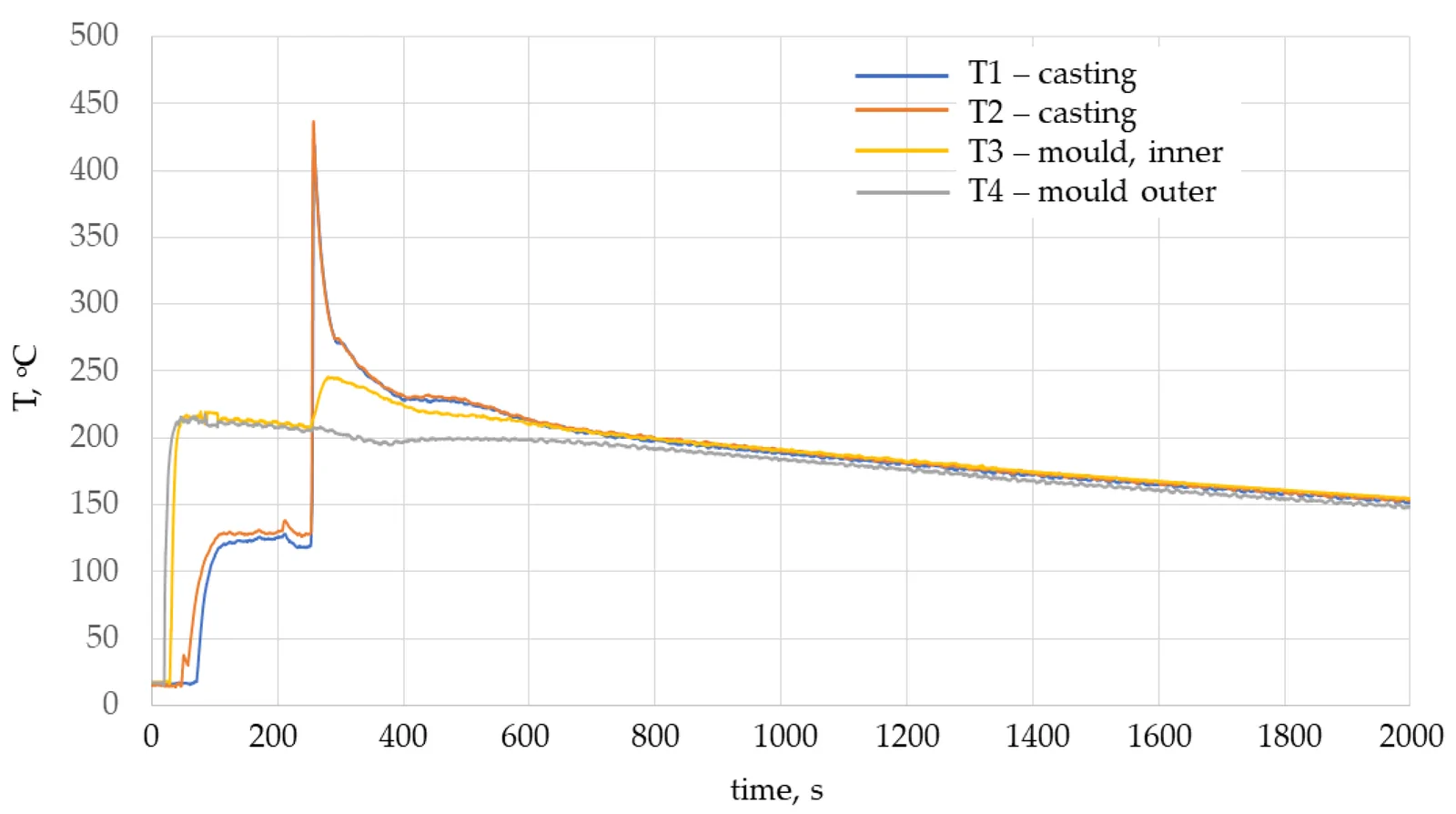
Thermal curves for the V738 alloy during centrifugal casting in a preheated cast-iron mould.

According to the thermal diagram, the initial temperature of the mould ring decreased from 300 °C in the furnace to 160–170 °C after the ring was mounted on the rotating table. The high thermal conductivity of the low-carbon, ferritic steel ring, combined with external water-mist cooling, resulted in rapid heat extraction and highly dynamic thermal conditions. The temperature became nearly uniform throughout the casting–mould system. The cooling rate was estimated at approximately 0.1 °C/s (6 °C/min) during the initial stage, when water-mist cooling was applied, and approximately 0.03 °C/s (1.8 °C/min) during the subsequent stage without water-mist cooling. Analysis of the cooling curves and their derivatives revealed that the thermal effects were less distinct and more dispersed than those observed in conventional TDA cups. No distinct thermal effect associated with the primary η phase was observed. The second transition was indicated only by an inflection point in the temperature curve, while the third appeared as a short plateau. The temperature associated with the formation of the second intermetallic phase, SnSb (β phase), was lower than under static conditions: 266–267 °C compared with 280–285 °C. Matrix formation was indicated at 227–229 °C, which was also below the corresponding temperature under static conditions (240–242 °C). Figure 9 presents the corresponding data obtained using the cast-iron mould.

**Figure 10:**
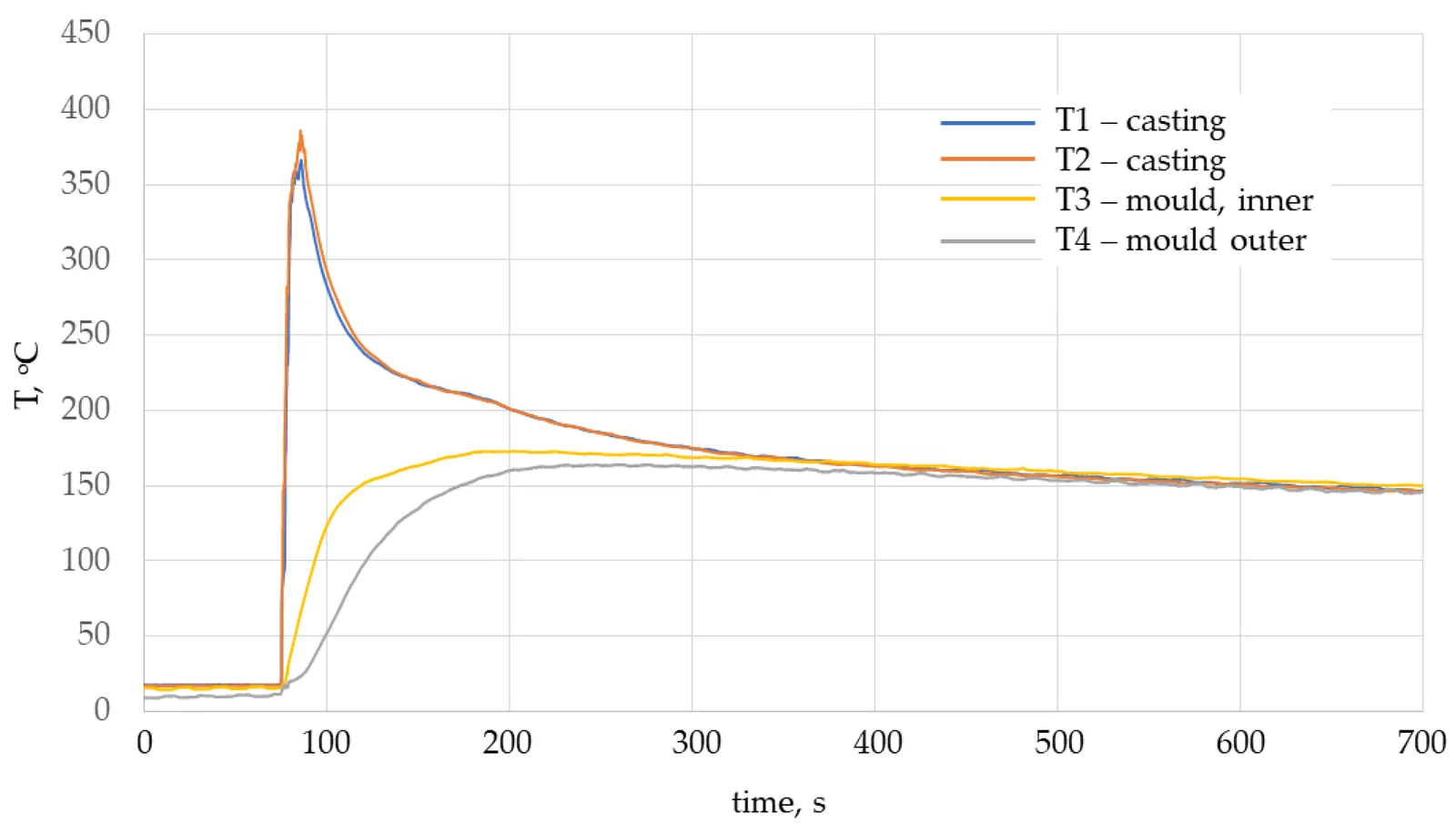
Thermal curves for the V738 alloy during centrifugal casting in a steel mould that is not preheated.

**Figure 11:**
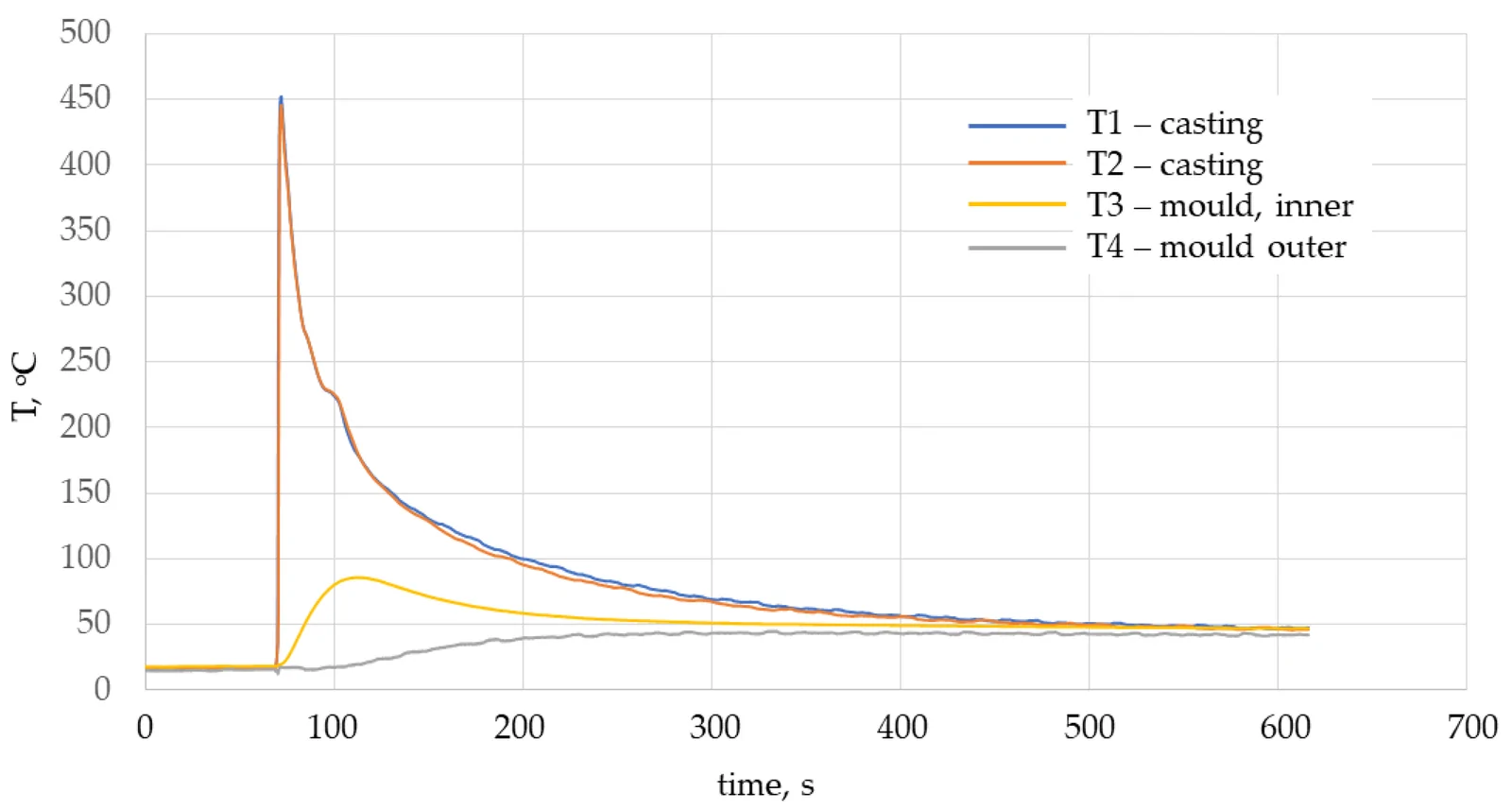
Thermal curves for the V738 alloy during centrifugal casting in a cast-iron mould that is not preheated.

In the case of the preheated cast-iron mould, the conditions were more stable, and some thermal effects were sufficiently distinct to allow analysis. The crystallisation curve still showed no clear indication of the leading phase. However, the thermal effect associated with the β phase was clearly visible, as was that associated with matrix crystallisation. In both cases, the corresponding temperatures were lower than those observed under static conditions. The thermal effects remained dispersed, although to a lesser extent than in the case of the preheated steel mould. The heat capacity associated with the mould geometry and the thermophysical properties of the mould material resulted in a smaller decrease in the initial temperature, from 300 °C to 210–220 °C, before pouring. The cooling rate was estimated at 0.17 °C/s (10 °C/min) during water-mist cooling and 0.045 °C/s (2.7 °C/min) during the subsequent stage without water-mist cooling. Figure 10 and Figure 11 show the thermal curves obtained using the same mould without preheating.

An analysis of the diagrams obtained using the steel mould shows that neither of the thermal effects associated with the intermetallic phases can be clearly distinguished. These effects are dispersed throughout the crystallisation curve. The final thermal effect, associated with matrix crystallisation, alters the shape of the cooling curve, providing a clear indication of the end of solidification. Interestingly, in the case of the cold cast-iron mould, the thermal effects associated with both the β phase and matrix crystallisation can be identified even in the cooling curve. In all cases, the characteristic temperatures are lower than those observed under static conditions. A more advanced analysis of the data, using higher-order derivatives, enabled the determination of the beginning and end of solidification for all four castings. The corresponding values and their ratios are presented in Table 4.

**Figure 12:**
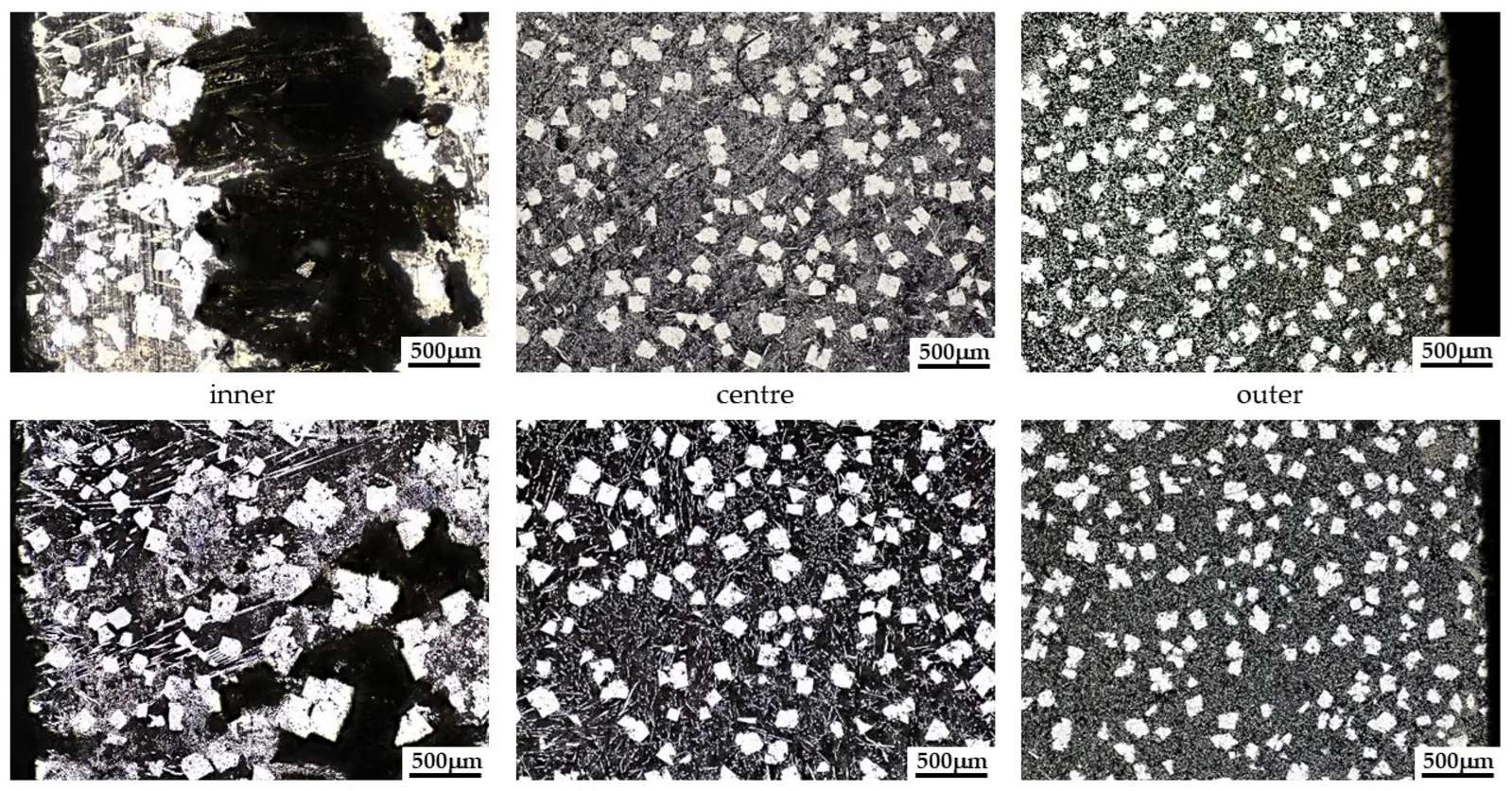
Microstructure of the V738 alloy cast in a preheated steel mould in centrifugal casting (mag. ×100, etched).

**Figure 13:**
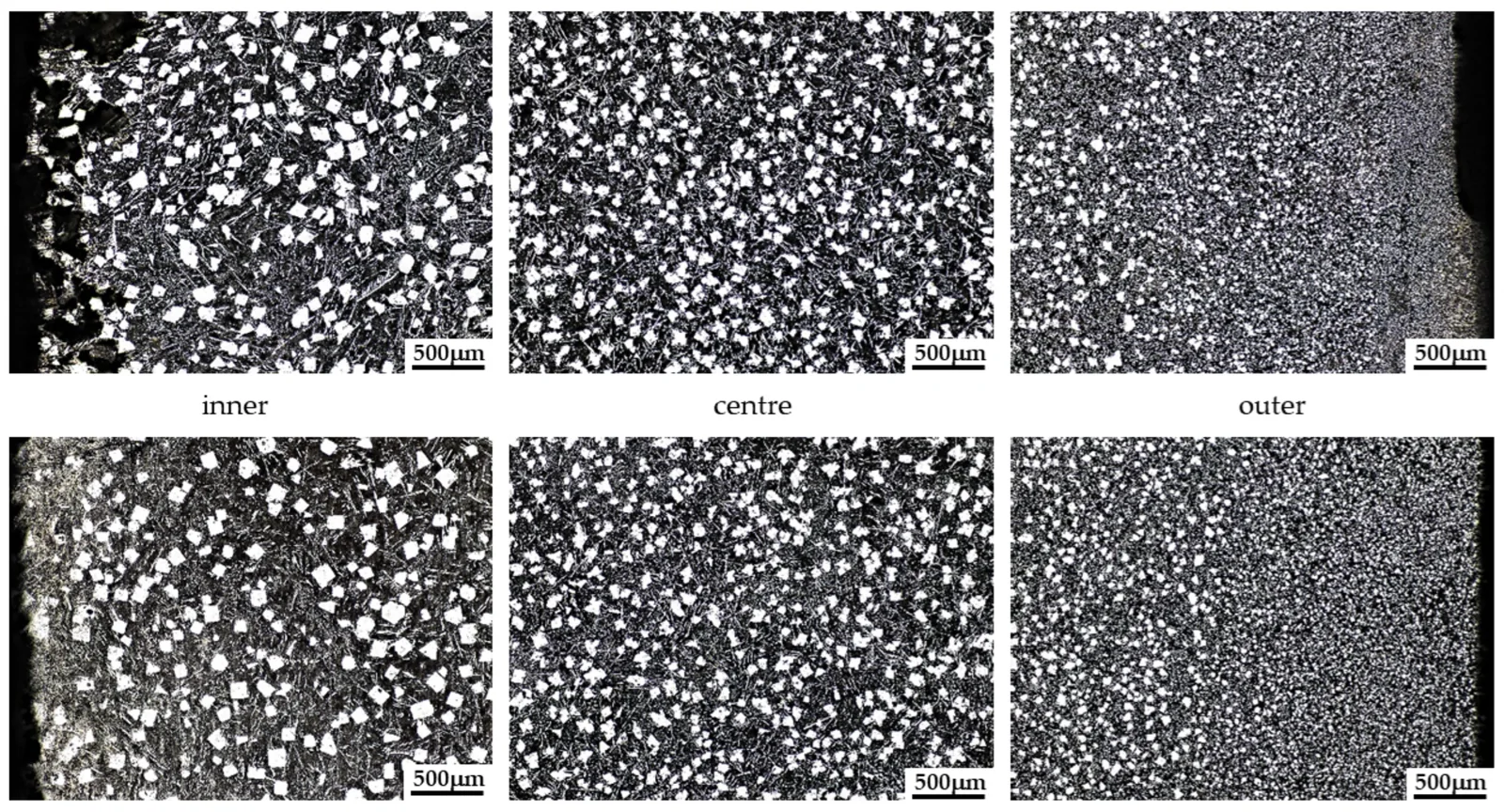
Microstructure of the V738 alloy cast in a cold steel mould in centrifugal casting (mag. ×100, etched).

**Figure 14:**
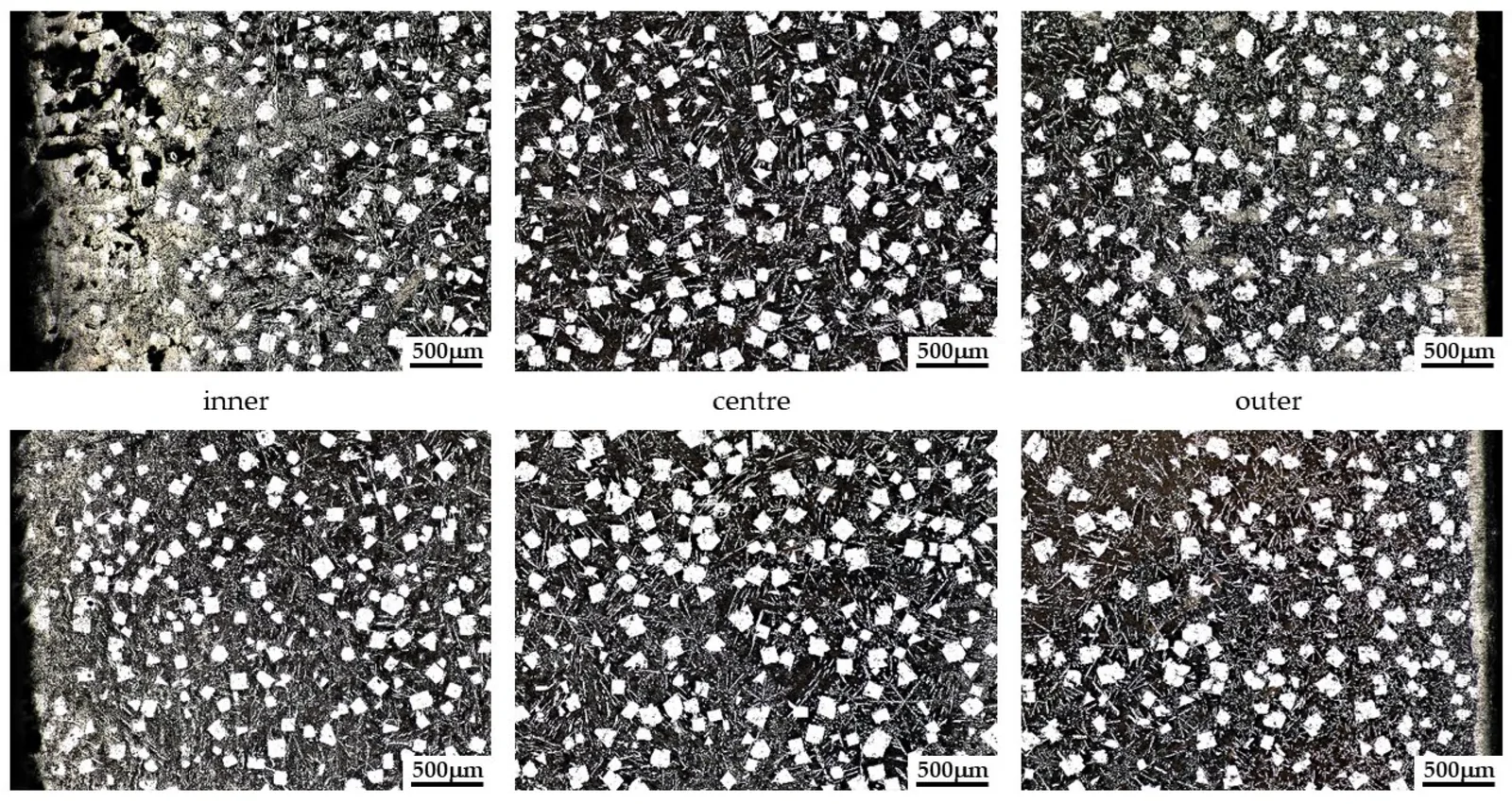
Microstructure of the V738 alloy cast in a preheated cast-iron mould in centrifugal casting (mag. ×100, etched).

**Figure 15:**
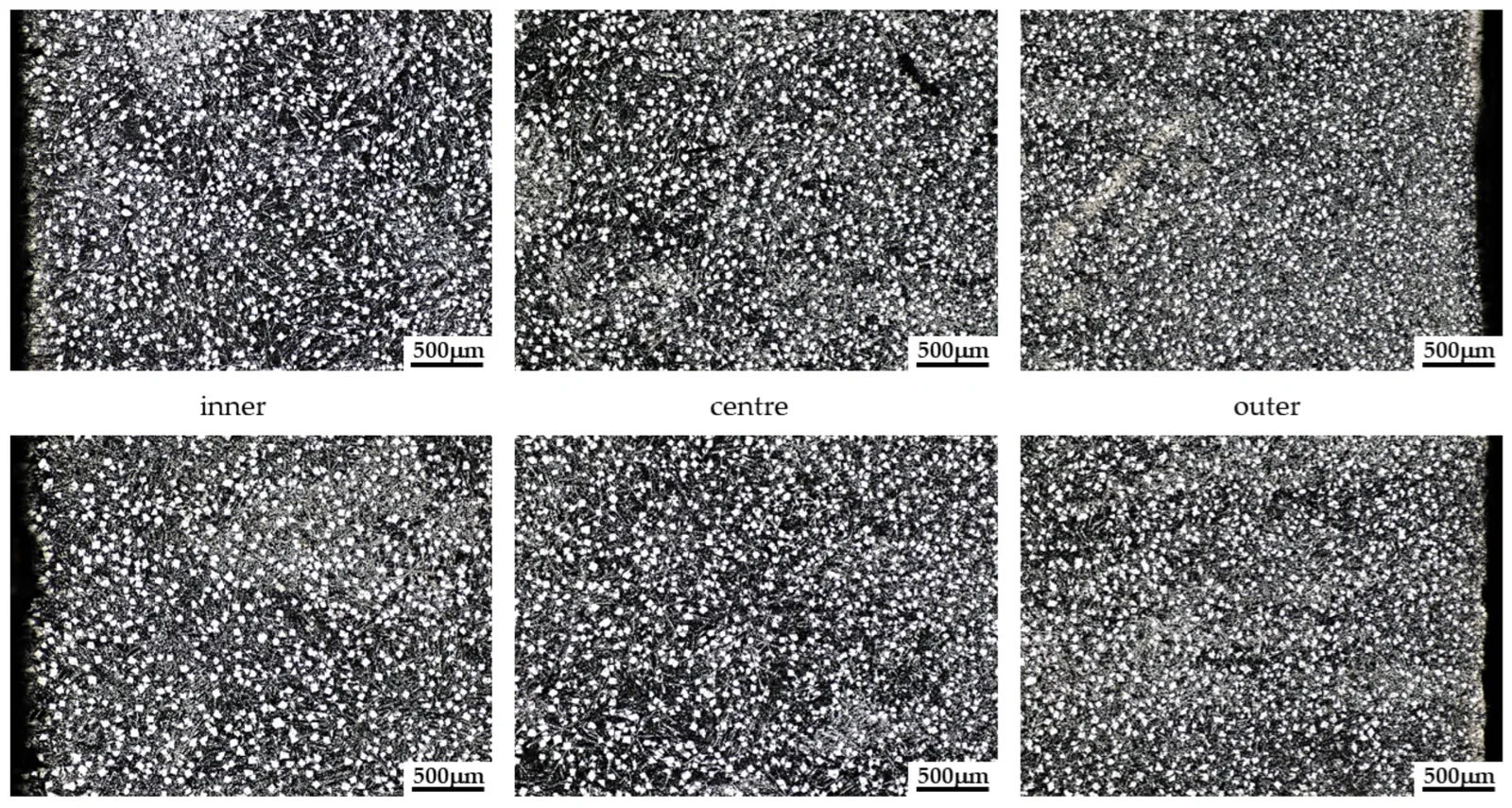
Microstructure of the V738 alloy cast in a cold cast-iron mould in centrifugal casting (mag. ×100, etched).

**Figure 16:**
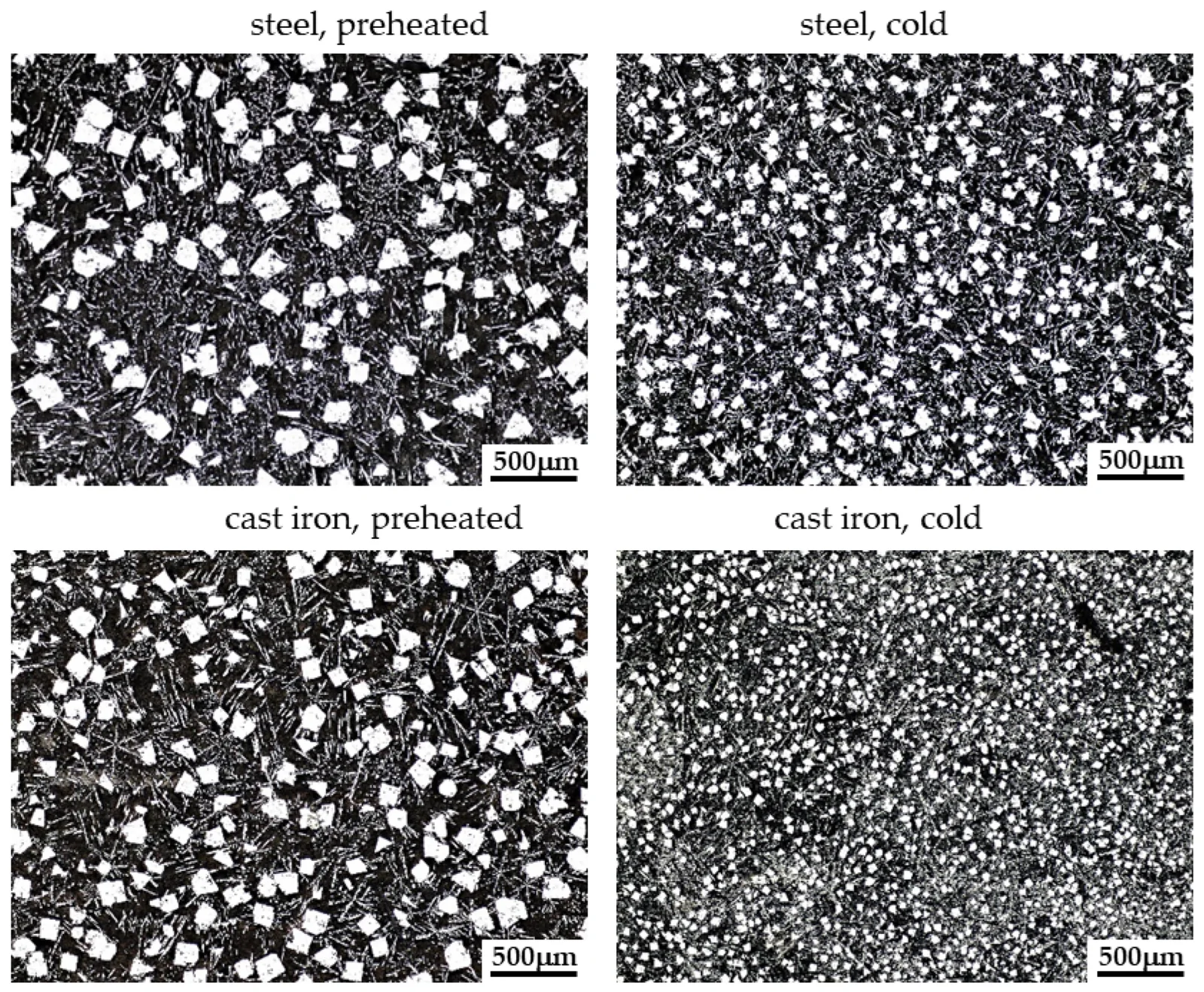
Overall comparison of microstructure for the V738 alloy cast in moulds at different states (mag. ×100, etched).

**Figure 17:**
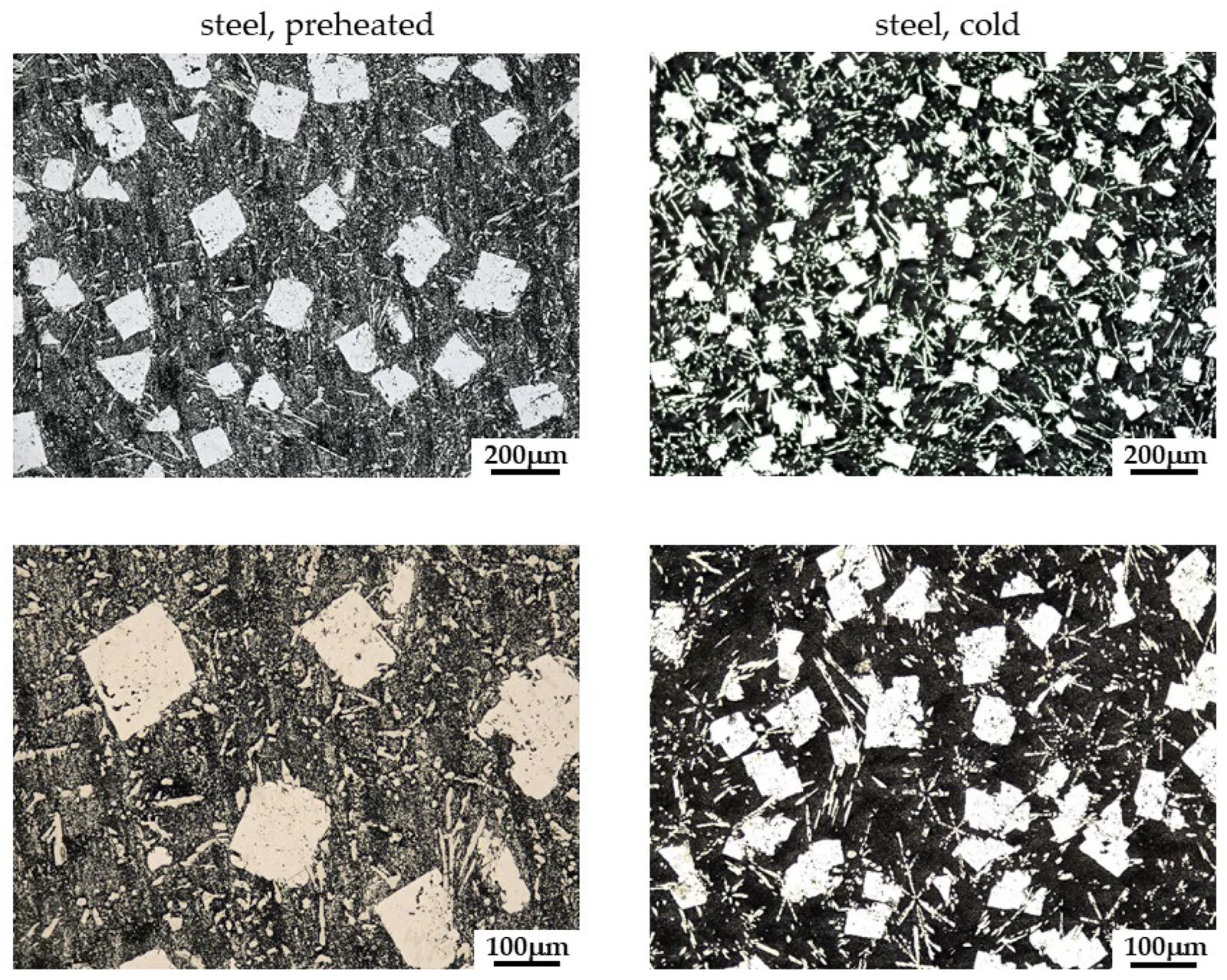
Microstructure of the V738 alloy cast in a steel mould in centrifugal casting (mag. ×200, ×400, etched).

**Figure 18:**
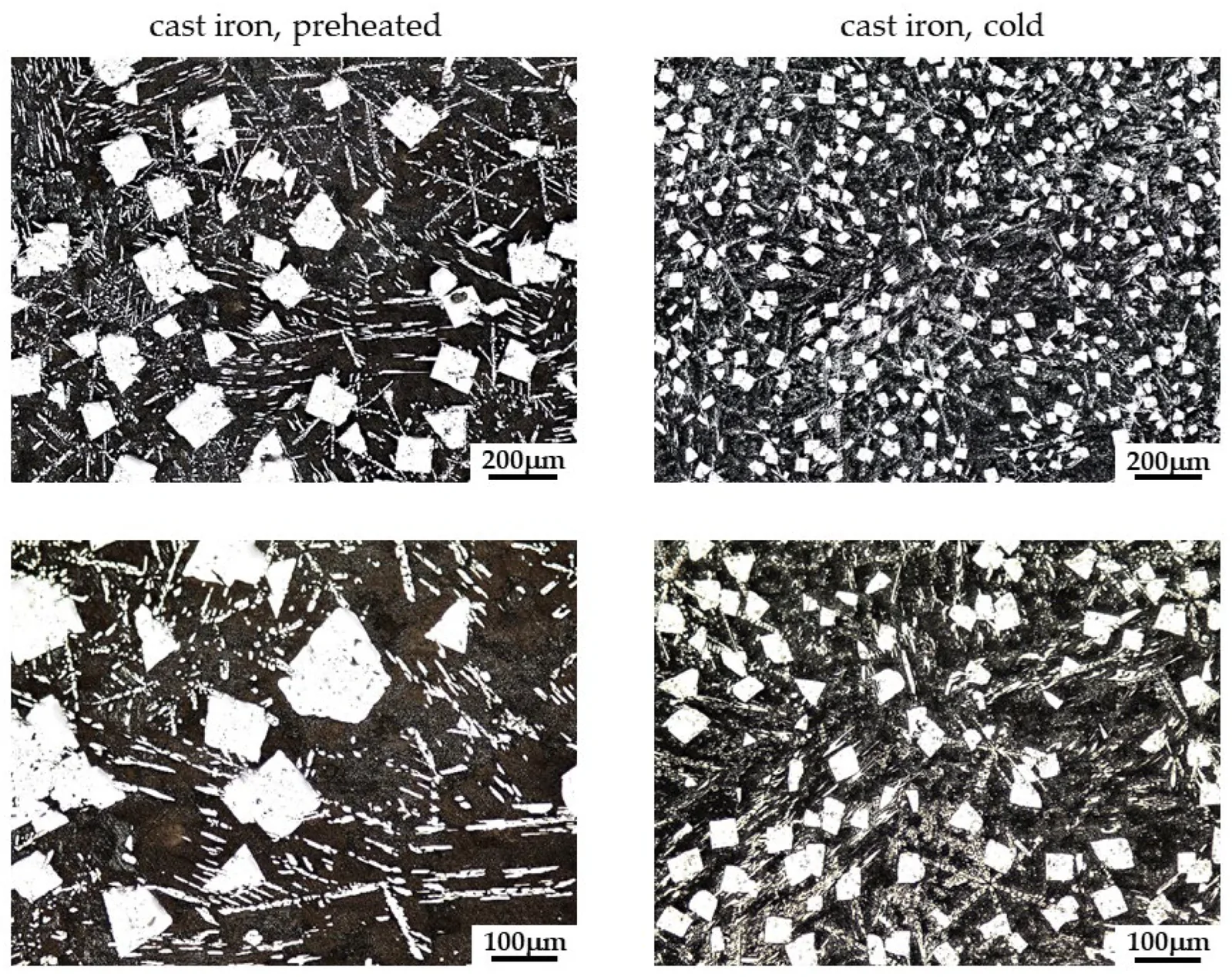
Microstructure of the V738 alloy cast in a cast-iron mould in centrifugal casting (mag. ×200, ×400, etched).

The next part of the experiment involved microstructural analysis. Figure 12, Figure 13, Figure 14, Figure 15, Figure 16, Figure 17 and Figure 18 present the main results obtained during centrifugal casting under different experimental conditions, including variations in mould material and preheating. The same methodology was applied to all sets of micrographs. The microstructure was examined in the middle section of the casting, covering both the mid-upper and mid-lower regions. Within each region, three locations across the cross-section were analysed: the inner, central, and outer parts. The inner part was located near the free surface of the casting, whereas the outer part was in contact with the mould cavity. This approach was adopted because of a simplifying assumption made in the experimental setup: the casting was treated as part of a larger ring, with its axial boundaries insulated by refractory material, thereby limiting heat extraction in the axial direction.

In both cases, the microstructure is refined near the outer part of the casting. Towards the middle of the cross-section, the β-phase crystals increase in size, with the largest crystals observed near the inner surface. This phenomenon can be attributed to variations in the heat-extraction rate during crystallisation. The higher cooling rate during the initial stage of the process promotes microstructural refinement. This effect is particularly evident in the case of the cold mould, where distinct regions can be observed in the outer part of the casting. The width of this region increases towards the lower part of the casting. Greater porosity, mainly resulting from shrinkage, is observed in castings produced using the steel mould. In the case of the preheated mould, the final shrinkage cavity is located near the inner surface (Figure 12).

Figure 14 and Figure 15 show the microstructures obtained using cast-iron moulds in the preheated and cold states, respectively. Differences in the geometric characteristics and thermophysical properties of the moulds resulted in microstructural variations compared with those observed in the steel mould. In the case of the preheated cast-iron mould, a distinct matrix layer is present near the outer surface of the casting. The size of the primary crystals (β phase) is relatively uniform throughout the cross-section, with no pronounced local variations. Shrinkage porosity is observed only in the upper part of the casting, near the inner surface, when the preheated mould is used. In contrast, the casting produced using the cold cast-iron mould exhibits the most uniform and refined microstructure among all the investigated conditions.

Figure 16, Figure 17 and Figure 18 present a series of comparative micrographs illustrating the influence of the applied casting conditions on microstructure formation. All micrographs were obtained from comparable regions of the castings and are presented at the same magnification.

As can be seen, microstructural uniformity, distribution, and refinement improve with increasing heat-extraction rates. Moreover, the use of a massive mould (cast iron) produces better results than the use of a steel mould, despite the latter’s higher thermal conductivity and the application of external cooling.

**Figure 19:**
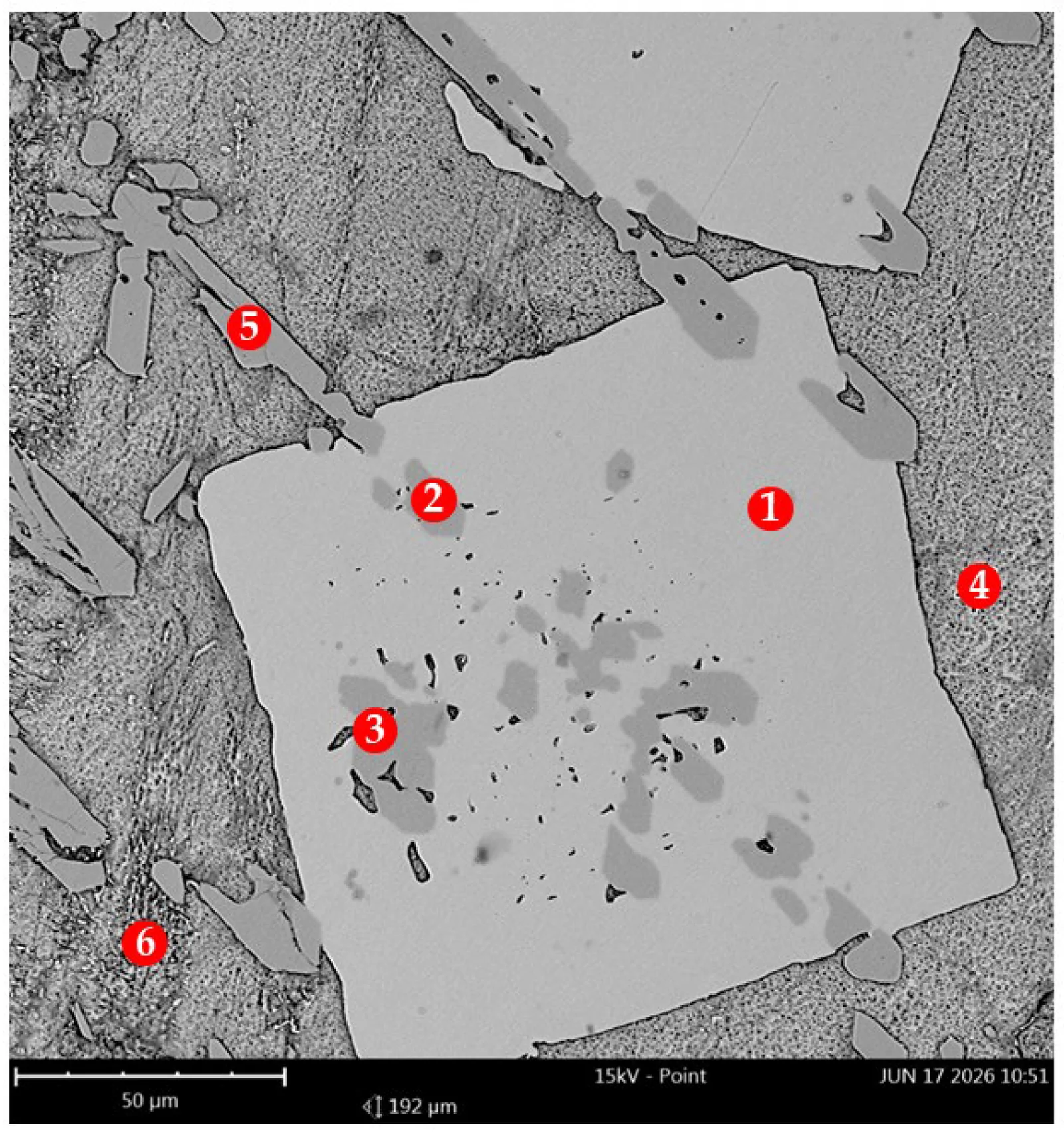
Microstructure of the V738 alloy with indicated points of EDS analysis, chemical composition is given in Table 5 (wt.%) and Table 6 (%at.).

**Figure 20:**
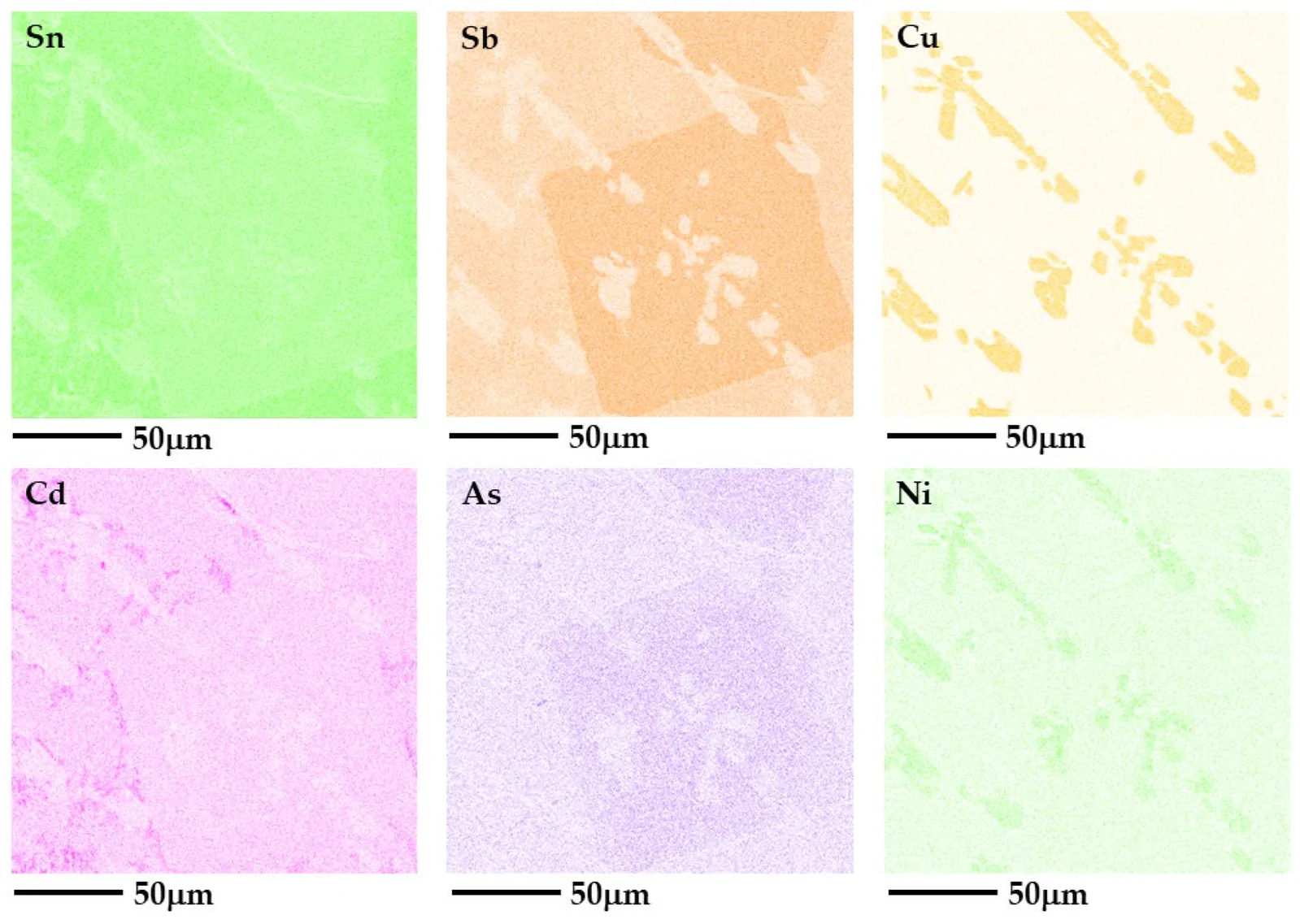
EDS map analysis—the element distribution across the microstructural components—of the region studied in Figure 19.

The influence of cooling conditions on the crystallisation of the primary η phase is similar. An increased heat-extraction rate leads to a reduction in crystal size and restricts the growth of secondary branches. The initial identification of the phases and the analysis of elemental distributions in different microstructural components were performed using scanning electron microscopy (SEM) and energy-dispersive X-ray spectroscopy (EDS). Figure 19 and Table 5 and Table 6 present the results of the point analyses. Figure 20 shows elemental maps illustrating the distribution of the chemical elements across the different microstructural components.

The primary η phase, which forms rosettes with needle-like arms, consists mainly of copper and tin. Nickel is also present and appears to be concentrated within these crystals. According to numerical calculations, some antimony may dissolve in the η phase, substituting for copper, with a calculated content of up to 3 wt.% at 300 °C. The measured atomic concentrations support this possibility, showing elemental proportions similar to those reported for the Cu_6_Sn_5_ intermetallic phase, although the experimentally determined values differ from the calculated ones. The β phase, which is the most prominent and structurally significant microstructural constituent, consists mainly of tin, antimony, and arsenic, while the nickel and cadmium contents are very low. The matrix consists predominantly of a solid solution of antimony in tin, with cadmium locally detected, particularly in the vicinity of both intermetallic phases. The qualitative distribution of the elements is illustrated by the elemental maps in Figure 20. These maps provide further insight into the distribution of the alloying additions and help clarify their potential roles. Nickel is concentrated mainly in the primary η phase. Its presence is associated with an increase in the initial temperature indicated by the TDA diagrams (liquidus temperature) and with a tendency towards more developed crystal morphology, including secondary arms, under static conditions and at low cooling rates. Arsenic is concentrated mainly in the β phase and contributes to increased hardness, whereas cadmium may inhibit the growth of both intermetallic phases.

## 4. Discussion

The results presented in this study encompass a wide range of data and several factors influencing the solidification process. The main objective was to qualitatively describe the relationships between the investigated parameters and identify the direction of their influence. As the primary focus of the paper is the solidification sequence, the discussion is limited to the relationships between technological parameters, the solidification process, and the resulting microstructure. The initial analysis was based on Thermo-Calc simulations, which served as a reference for interpreting the experimental results. For several reasons, the chemical composition used in the calculations was simplified to the three principal elements: Sn, Sb, and Cu. This composition corresponds to the basic SnSb11Cu6 alloy and provides an equilibrium reference for the experimental results, against which the influence of additional alloying elements in V738 can be assessed. This approach also makes it possible to interpret the effects of the additional elements in V738 in relation to the reference SnSb11Cu6 alloy. The simulations established the solidification sequence of the model alloy under equilibrium conditions. Comparison with the experimental results obtained under conditions closest to equilibrium—that is, for the SnSb11Cu6 alloy poured into a sand cup—revealed the same sequence of phase formation. However, the initial temperature observed experimentally was significantly higher than the calculated value (360 °C and 300 °C, respectively). Interestingly, the differences between the calculated and experimentally determined temperatures of the subsequent transitions were much smaller. This suggests that the η phase has a high nucleation potential even under non-equilibrium conditions. As the conditions for the SnSb11Cu6 alloy departed further from equilibrium, particularly when cast-iron cups were used in preheated and unheated conditions and the impurity content increased, the Cu_6_Sn_5_ intermetallic phase proved to be the most sensitive to these changes. Under some conditions, its associated thermal effect became indistinct in the TDA curves. At higher cooling rates, the use of second- or third-order derivatives was necessary to identify the temperature range associated with this transition. In such cases, the heat effect was distributed along the cooling curve, changing its slope or producing an inflection point rather than a distinct peak. Microstructural examination showed refinement of all phases, accompanied by a reduction or even the absence of secondary branches in the η-phase crystals. In static casting, the other two thermal effects, associated with the formation of the SnSb (β) phase and the matrix, were less sensitive to increased cooling rates. The second alloy investigated, V738, exhibited the same solidification sequence and similar trends, although the phase transitions occurred at higher temperatures than in the reference SnSb11Cu6 alloy. The higher onset temperature of the primary η phase may be associated with the presence of nickel, as indicated by the microanalytical results. Arsenic was found predominantly in the β phase, whereas cadmium was detected mainly in the matrix, with elevated concentrations in the regions surrounding the η- and β-phase crystals. The alloying additions are expected to contribute to increased hardness at both room and elevated temperatures. In the present study, the hardness of V738 was approximately 20% higher than that of SnSb11Cu6, increasing from 30 HB to 36 HB (2.5 mm ball, 62.5 kgf load; authors’ own measurements). The microhardness was also higher in V738. For the β phase, the values were 101 HV and 125 HV for SnSb11Cu6 and V738, respectively, while the corresponding matrix values were 30 HV and 35 HV (authors’ own measurements, 50 gf load).

The next step towards increasingly non-equilibrium conditions was to investigate solidification during centrifugal casting in preheated and unheated moulds. Depending on the initial mould temperature and its thermophysical and geometric characteristics, the thermal effects associated with individual phase transitions could be difficult to distinguish. Interestingly, the cooling rate alone did not determine the resulting microstructural quality, as demonstrated by the differences between the steel and cast-iron moulds. The massive cast-iron mould, owing to its capacity to absorb and store heat and its ability to extract heat under the conditions investigated, produced a more favourable microstructure. This observation is consistent with industrial practice, in which thin-walled steel bearing housings can be more challenging to cast and may exhibit defects such as porosity, incomplete bonding, and segregation. The TDA curves obtained during centrifugal casting were less distinct than those recorded under static conditions. The characteristic transitions were detected at lower temperatures than in conventional TDA measurements. The formation of the matrix at the final stage of solidification was the least affected in terms of its detectability. For all transitions, the thermal effects associated with latent heat release were distributed along the cooling curves, modifying their slopes rather than producing clearly defined peaks. Among the investigated conditions, centrifugal casting in the unheated cast-iron mould resulted in the most uniform and refined microstructure. Conversely, the poorest structural quality was observed in the preheated steel mould, where shrinkage-related defects were particularly pronounced. Despite the differences in the recorded solidification behaviour, the overall phase composition of the resulting microstructures remained similar. The main differences concerned phase morphology, size, and distribution. Higher cooling rates and centrifugal rather than static casting promoted phase refinement, particularly of the β-phase crystals, and altered the morphology of the η phase. The matrix also changed from a relatively simple solid-solution structure to a more eutectic-like morphology containing fine intermetallic crystals of both types.

## 5. Conclusions

The studies presented in this paper form part of a broader research project motivated by industrial needs. The initial experiments described in this paper provide a basis for further research. Nevertheless, several conclusions can be drawn at this stage regarding tin-based bearing alloys produced by static and centrifugal casting:

1. For the reference alloy SnSb11Cu6, the crystallisation sequence observed experimentally is consistent with the simulation results. The main differences concern the temperatures, which are higher in the experiments than those predicted by the simulations.
2. The addition of Ni, Cd and As increased the temperatures of all phase transitions without introducing additional thermal effects in the TDA curves or additional microstructural constituents.
3. An increased cooling rate positively affected the microstructure by refining the intermetallic phases. The smaller Cu_6_Sn_5_ and SnSb crystals resulted in a more uniform microstructural distribution. The TDA results indicated that the increased cooling rate altered the crystallisation process, making the thermal effects less distinct or shifting them to different temperatures.
4. Additional physical factors associated with centrifugal casting further alter the TDA curves, changing the characteristic temperatures of the β phase and the matrix and affecting the morphology of the intermetallic phases.
5. The investigation of model casting–mould systems designed to reproduce industrial conditions helped identify factors that may explain the main differences between plain bearing castings and the defects that occur when casting into steel and cast-iron housings.

## Figures and Tables

**Table 1:** Chemical composition of the alloys used in the studies; values given in wt.%.

Type	Sn	Cu	Sb	As	Cd	Ni	Fe	Pb	Other Σ
SnSb11 Cu6	Balance	5.0	11.6	0.0005	0.001	0.002	0.008	0.033	0.007
V738	Balance	5.2	12.11	0.51	1.36	0.24	0.006	0.013	0.010

**Table 2:** Geometric characteristics of the moulds and castings used in the centrifugal casting experiments.

Type	D1 mm	D2 mm	D3 mm	Thickness mm	Mould kg	Casting kg
Steel	210	250	320	35	8.59	3.58
Cast iron	106	128	296	35	13.68	0.95

**Table 3:** Crystallisation sequence calculated using Thermo-Calc software, showing the characteristic points and phase fractions at each stage of solidification.

Composition	Temperature Range	Liquid %	η %	β %	α %
Liquid	510 → 300	100	0	0	0
Liquid + η	300 → 260	100 → 94	0 → 6	0	0
Liquid + η + β	260 → 230	94 → 72	6 → 14	0 → 14	0
Liquid + η + β + α	230 → 230	72 → 63	14 → 23	14	0
Liquid + η + β + α	230 → 230	63 → 0	23	14	0 → 63
η + β + α	230 → 20	0	23 → 13	14 → 27	63 → 60

**Table 4:** Comparison of solidification times for centrifugal castings.

Type	Preheated tp, s	Cold tc, s	Ratio tp/tc
Steel	406	93	4.4
Cast iron	252	29	8.7

**Table 5:** EDS point analysis of the wt.% composition for different phases in the structure of the V738 alloy under centrifugal casting in a preheated cast-iron mould.

Element Point	wt.%					
	Sn	Sb	Cu	Cd	Ni	As
1	56.34	39.07		0.72	0.36	3.51
2	58.93	8.72	27.01	0.81	3.91	0.62
3	67.67	24.53	3.73	4.07		
4	72.97	18.93		8.10		
5	59.48	8.42	29.14	1.32	1.64	
6	74.98	21.19		3.84		

**Table 6:** EDS point analysis of the at. composition for different phases in the structure of the V738 alloy under centrifugal casting in a preheated cast-iron mould.

Element Point	% at.					
	Sn	Sb	Cu	Cd	Ni	As
1	55.52	37.53		0.75	0.72	5.48
2	46.17	6.66	39.54	0.67	6.19	0.77
3	65.79	23.26	6.78	4.17		
4	72.99	18.46		8.55		
5	46.89	6.47	42.92	1.10	2.62	
6	75.21	20.72		4.06		

## Data Availability

The original contributions presented in this study are included in the article. Further inquiries can be directed to the corresponding author.
